# Saccharification and liquefaction of cassava starch: an alternative source for the production of bioethanol using amylolytic enzymes by double fermentation process

**DOI:** 10.1186/1472-6750-14-49

**Published:** 2014-05-29

**Authors:** Sidra Pervez, Afsheen Aman, Samina Iqbal, Nadir Naveed Siddiqui, Shah Ali Ul Qader

**Affiliations:** 1 The Karachi Institute of Biotechnology & Genetic Engineering (KIBGE), University of Karachi, Karachi -75270, Pakistan

**Keywords:** Amylases, *Aspergillus fumigatus*, Biofuel, Saccharification, *Saccharomyces cerevisiae*, Starch

## Abstract

**Background:**

Cassava starch is considered as a potential source for the commercial production of bioethanol because of its availability and low market price. It can be used as a basic source to support large-scale biological production of bioethanol using microbial amylases. With the progression and advancement in enzymology, starch liquefying and saccharifying enzymes are preferred for the conversion of complex starch polymer into various valuable metabolites. These hydrolytic enzymes can selectively cleave the internal linkages of starch molecule to produce free glucose which can be utilized to produce bioethanol by microbial fermentation.

**Results:**

In the present study, several filamentous fungi were screened for production of amylases and among them *Aspergillus fumigatus* KIBGE-IB33 was selected based on maximum enzyme yield. Maximum α-amylase, amyloglucosidase and glucose formation was achieved after 03 days of fermentation using cassava starch. After salt precipitation, fold purification of α-amylase and amyloglucosidase increased up to 4.1 and 4.2 times with specific activity of 9.2 kUmg^-1^ and 393 kUmg^-1^, respectively. Concentrated amylolytic enzyme mixture was incorporated in cassava starch slurry to give maximum glucose formation (40.0 gL^-1^), which was further fermented using *Saccharomyces cerevisiae* into bioethanol with 84.0% yield. The distillate originated after recovery of bioethanol gave 53.0% yield.

**Conclusion:**

An improved and effective dual enzymatic starch degradation method is designed for the production of bioethanol using cassava starch. The technique developed is more profitable due to its fast liquefaction and saccharification approach that was employed for the formation of glucose and ultimately resulted in higher yields of alcohol production.

## Background

Emerging environmental issues raised due to combustion of petroleum-based fossil fuel and emission of toxic gases have diverted the attention of scientists and researchers towards the utilization of various renewable resources for the production of bioethanol. In addition to these global concerns, other important factors that have been kept in preference are the mounting prices of the fuels and the current political scenario among the oil producing nations. Bioprocessing of renewable resources available in a particular region can help in resolving these issues. Various renewable resources in terms of agricultural biomass have been investigated for the production of bioethanol and this development proved beneficent for the biotechnological industries. Amongst various starchy materials available throughout the world; corn, sugarcane, wheat, potato [1], corn stover [2,3], molasses [4] and purified starch [5] have been successfully utilized for the commercial production of bioethanol. As the demand and the cost of these starchy crop materials is increasing day by day, it has become indispensible to use substitute raw resources.

Cassava is a tropical root crop which is an economically available fermentable source and is produced by numerous countries [6]. It is incorporated into animal feed (20.0%) and about similar proportion is converted into starch for industrial purposes whereas; some of the portion is also used as food source in several developing countries. About 50.0 to 70.0% starch content is recovered from the cassava root and due to the low ash content and rich organic nature it can be used as an ideal substrate for bioethanol production [7-9]. In addition, it can also be easily hydrolyzed by various techniques. As cassava starch does not have much industrial application in food industries as compared to corn starch, therefore it also lacks competition in terms of price and is available throughout the year due to its flexibility in terms of planting and harvesting [7,10,11].

In recent years, bioprocessing of various value-added products using microbial factories have been potentially explored with reference to extracellular enzymes. Agricultural biomass used as a substrate for the production of bioethanol has several limitations including high fiber content which requires high temperature for hydrolysis and this energy intensive procedure also does not provide desired yields of fermentable sugars. Hydrolysis of lingocellulosic mass by other expensive pre-treatment techniques is also time consuming. Industries also have concerns regarding the availability of the biomass throughout the year and most of the time its storage in bulk quantities is not possible due to space shortage. The development of an ideal pre-treatment method for hydrolyzing poly-phenolic lignin in the feedstock is expensive with several aforementioned limitations thus, enzymatic treatment is more preferable. Conventional method used for the production of bioethanol from cassava starch usually requires the basic gelatinization step followed by liquefaction and saccharification. The sugar formed during these processes is further fermented using either yeast or bacteria. Since, starch derived from any plant source is a complex molecule, it require various hydrolytic enzymes for its conversion into simple fermentable sugars. Among many extracellular hydrolases available, microbial amylases are frequently used for its conversion. For commercial production of amylases *Aspergillus* and *Rhizopus* species are considered most significant sources because the enzymes from these sources are generally thermostable and are available in excessive quantities [12-14].

Despite several advantages of simultaneous saccharification and fermentation using multiple organisms, there are also few shortcomings. In the initial steps, the amylolytic enzymes are produced using fungi and the starch present in the medium is allowed to hydrolyze into simpler sugars and afterwards another microbial factory (yeast or bacteria) is incorporated in the same fermentation flask to produce ethanol. In this case the primary organism (specifically fungal specie) along with amylolytic enzymes also excretes other toxic substances and proteases which in result inhibit the growth and performance of the second ethanol-producing microorganism. Along with this, the establishment of appropriate temperature for starch hydrolysis, enzymatic activity and ethanol production also plays an important role. Current research deals with the production of bioethanol from hydrolysis of an inexpensive renewable resource known as cassava starch, which is commonly available in Pakistan. The methodology used for the production of ethanol was based on double fermentation technique using partially purified fungal amylolytic enzymes for the liquefaction and saccharification of this starchy material. Keeping all disadvantages in view, this study was designed in two separate steps. In first step, amylolytic enzymes (α-amylase and amyloglucosidase) were produced using indigenously isolated filamentous fungi and were partially purified to hydrolyze cassava starch into simple fermentable sugars. In the next step, the sugar cocktail was fermented using *S. cerevisiae* to acquire maximum bioethanol yield.

## Results and discussion

In the present study, several different fungal isolates with amylolytic activities were purified from different soil samples and preliminary identification was based on microbiological studies including cultural and microscopic characterization followed by 18S rDNA sequence analysis. Colonial and microscopic characteristics indicate that all isolates belong to genera *Aspergillus*. Microscopic morphology of *A. fumigatus* KIBGE-IB33 showed columnar and uniseriate conidial heads while, conidiophores are short and smooth. On the other hand, *A. niger* KIBGE-IB36 showed large, globose, dark brown conidial heads with hyaline and smooth conidiophores. Likewise, conidial heads of *A. flavus* KIBGE-IB34 are radiate and biseriate whereas, conidiophores are hyaline and coarsely roughened. *A. terreus* KIBGE-IB35 has biseriate and globose conidia with hyaline and smooth conidiophores. *A. versicolor* KIBGE-IB37 showed centrally rising, velvety floccose and slightly blue-green color colony on PDA with conidiophores borne from surface or aerial hyphae.

**Figure 1:**
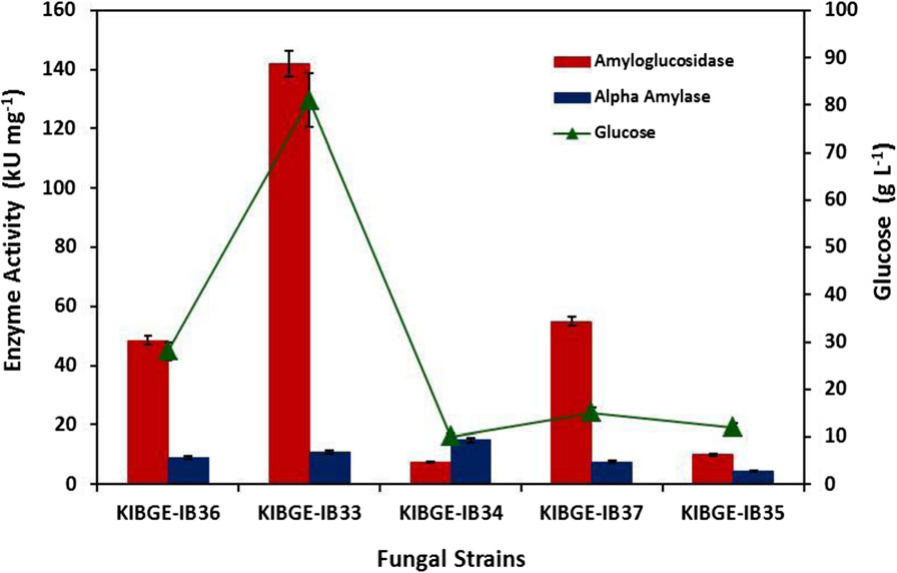
**Production of amylolytic enzymes and glucose formation by various filamentous fungi.** KIBGE-IB33: *A. fumigatus*; KIBGE-IB34: *A. flavus*; KIBGE-IB35: *A. terreus*; KIBGE-IB36: *A. niger*; KIBGE-IB37: *A. versicolor*.

Screening of amylolytic property of the strains was based on starch hydrolysis method. Initially, 07 fungal strains were selected and among them 05 filamentous fungi including *A. fumigatus* KIBGE-IB33*, A. flavus* KIBGE-IB34*, A. terreus* KIBGE-IB35*, A. niger* KIBGE-IB36 and *A. versicolor* KIBGE-IB37showed production for amylolytic enzymes. When these isolates were cultivated in the starch containing production medium, highest titers of α-amylase (11.0 kUmg^-1^) and amyloglucosidase (142.0 kUmg^-1^) were produced by *A. fumigatus* KIBGE-IB33 (Figure 1). This strain was also capable of producing considerable amount of glucose (81.0 gL^-1^) which can be used for the production of ethanol. The fermentable sugar produced by this isolate can be easily metabolized by *S. cerevisiae*. However, other filamentous fungi produced lower titers of both α-amylase and amyloglucosidase along with lower concentration of glucose. Although maximum α-amylase was produced by *A. flavus* KIBGE-IB34 but it did not showed higher glucose formation rate as compared to *A. fumigatus* KIBGE-IB33 therefore, this isolate was selected for further studies.

**Figure 2:**
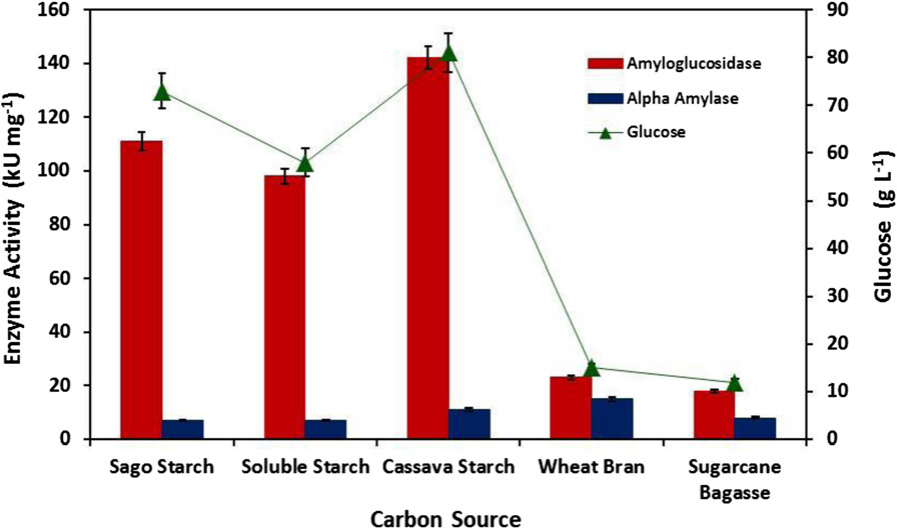
**Production of amylolytic enzymes from *Aspergillus fumigatus *** **KIBGE-IB33 and glucose formation using various carbon sources.**

**Figure 3:**
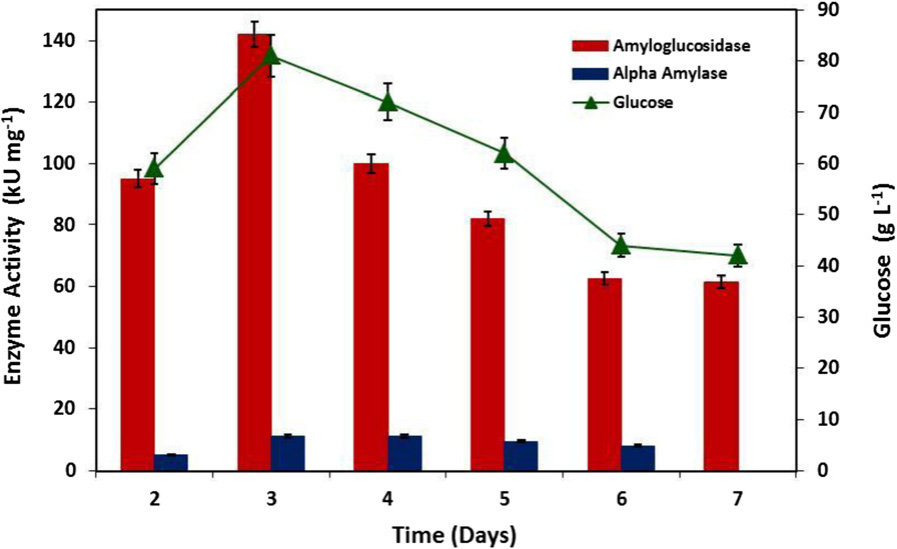
**Production of amylolytic enzymes and glucose formation by *Aspergillus fumigatus *** **KIBGE-IB33 at different incubation times.**

Optimization of various cultivation parameters plays an important role. For effective bioethanol production, fermentation time of the microbial culture and the type of the renewable carbon source used for the production of starch hydrolyzing enzymes and ethanol are among the most important factors. The aforementioned factors will ultimately direct the overall process cost for the development or the scale up of a new methodology in any bioethanol producing industry. Hence, fermentation time for the production of α-amylase and amyloglucosidase and different types of carbon sources were studied. Varieties of carbon source have been tested and can play an important role during microbial fermentation because they are the integral components for the production of cellular material and most of the time they are also associated with microbial growth [15]. Much interest has been diverted towards the utilization of economically available carbon sources in order to fulfill the industrial requirements. In the current study, to improve the production of α-amylase and amyloglucosidase for starch hydrolysis and bioethanol production, seven different carbon sources were utilized (Figure 2). The induction pattern for both amylolytic enzymes was different in various carbon sources suggesting that these hydrolases are inducible. Among all, cassava starch proved to be the most favorable inducer and contribute highest amount of enzyme units (α-amylase: 11.0 kUmg^-1^; amyloglucosidase: 142.0 kUmg^-1^) and glucose formation (81.0 gL^-1^) as compared to the other carbon sources tested. These results suggest that pure starch based carbon sources including sago starch, soluble starch (potato) and cassava starch are more suitable for the production of enzyme and glucose formation as compared to the different complex biomass (wheat bran and sugarcane bagasse) whereas, no enzyme production was detected when wheat starch and rice bran were used. This is because the lingocellulosic tough plant matrix was not pretreated. The cell free filtrate (CFF) collected after fermentation showed negligible titers for cellulase, pectinase and xylanase (data not shown) therefore; the starch content was not accessible for fermentation as compared to the purified starch materials. Fatima and Ali [16] tested sixteen fungal species for the production of amyloglucosidase (activity ranged between: 1.906-12.675 U ml^-1^ min^-1^) using starch in fermentation medium and the best strain they identified was *A. oryzae* llB-6 (12.673 ± 0.998 Uml^-1^ min^-1^). They also noticed a 30% increase in the enzyme activity when some of the process parameters were altered (pH and incubation time). Very recently, Puri *et al*. [17] reported the use of rice bran: wheat bran (1:1), rice bran: paddy husk (1:1) for the production of amylase and amyloglucosidase and the maximum amylase (2.72 IU) and amyloglucosidase (4.11 IU) activity was achieved when rice bran was incorporated in the fermentation medium inoculated with *A. oryzae*. However, *A. fumigatus* NTCC 1222 exhibited 341.7 U/mL amylase activity under solid state fermentation when incubated at 35°C (pH-6.0) for 06 days in nutrient salt solution [18]. In another study, detergent mediated production of glucoamylase in the presence of soluble starch is also reported using *A. niger* FME under shake flask system [19]. Several other researchers have also used cassava starch and cassava pulp as alternative carbon source for bioethanol production using α-amylase and amyloglucosidase [20-23]. The compositional analysis of cassava starch used in this study is presented in Table 1. The type and source of starch based materials plays a crucial role for achieving maximum bioethanol yield. The starch content in variety of biomaterials will govern the cost of bioethanol production. Therefore, before considering the production of bioethanol using a specific source, the nature of the starch molecule (linkage, granule size and shape) and the method of extraction employed must be kept in consideration. However, the values of compositional analysis of starch cannot be compared with other starch sources because of the variation of the plant source and the methods used to analyze the structure or content of starch. After selection of a suitable carbon source, fermentation time for the production of amylolytic enzymes was also studied by incubating *A. fumigatus* KIBGE-IB33 for different time interval ranging from 02 to 07 days. It was observed that production of α-amylase and amyloglucosidase started after 02 days of incubation and both the hydrolases were continuously produced up to day 06 and 07, respectively with a maximum titter secreted at day 03 (Figure 3). Afterwards, it was also noticed that as incubation time increases, amylolytic activity as well as glucose formation decreases. This might be due to the fact, with the passage of time the nutrients become depleted and other secondary metabolites are formed which eventually alters the pH of the medium and inhibits both the growth of the fungi as well as enzyme secretion [24]. Most of the time, secondary metabolites have a catabolic repression effect. Amylase from fungal sources is normally produced after 03 to 07 days of incubation but in some cases, enzyme secretion can be extended up to 15 days [25-28]. Prolong incubation time is one of the drawbacks of using filamentous fungi at industrial scale level which eventually increases process cost.

**Table 1:** Compositional analysis of commercially available cassava starch

**Composition**	**Cassava content (%, *w*/ *w *)**
Total sugar*	85.2 ± 4.26
Total protein*	1.4 ± 0.07
Reducing sugar*	6.2 ± 0.31
Glucose*	nil
Moisture content**	0.9 ± 0.04
Amylose***	10.7 ± 0.53
Amylopectin***	89.3 ± 4.46

*2.0% Cassava solution.

**1.0 g Cassava starch.

***0.4 g Cassava starch.

±Specifies standard deviation (SD) among three equivalent replicates. Values in each set differ significantly: p ≤ 0.05.

After fermentation, α-amylase and amyloglucosidase were partially purified from the CFF. Most of the time in CFF some other constitutive enzymes may also be present including various types of proteases which could cause interference during liquefaction and saccharification process of cassava starch. Therefore, to avoid any hindrance, starch hydrolyzing enzymes from the CFF were purified using gradient precipitation in the presence of ammonium sulfate ranging from 20.0% to 80.0% and among it 40% saturation level was selected. Table 2 summarizes the purification profile of α-amylase and amyloglucosidase. Fold purification of both α-amylase and amyloglucosidase increased up to 4.1 and 4.2 times with a specific activity of 9.2 kUmg^-1^ and 393 kUmg^-1^, respectively. Previously, Slivinski *et al.*[29] and da Silva and Peralta [30] have also reported precipitation of amyloglucosidase using ammonium sulfate produced by *A. niger* and *A. fumigatus*, respectively*.* Ammonium sulfate precipitation method was also used for purification of α-amylase from *Pencillium chrysogenum* and *A. niger* JGI 24, respectively [31,32]. The enzyme kinetic analysis showed that the optimum pH, temperature, V_max_ and K_m_ values for α-amylase from *A. fumigatus* KIBGE-IB33 were 6.0 (citrate buffer, 50.0 mM), 65°C, 25.0 kU ml^-1^ and 0.5 mg ml^-1^, respectively. Whereas, optimum pH, temperature, V_max_ and K_m_ values for amyloglucosidase were 5.0 (citrate buffer, 50.0 mM), 60°C, 105.0 kU ml^-1^ and 2.56 mg ml^-1^, respectively. The optimum pH and temperature for amyloglucosidase activity isolated from *A. niger* FME was 5.0 and 45°C, respectively whereas, the K_m_ and V_max_ values were determined using soluble starch as substrate as 94 μg ml^-1^ and 39.02 Umg^-1^, respectively [19]. In another recent study, the optimum pH of amyloglucosidase was 6.0 and the optimum temperature was 60°C along with K_m_ and V_max_ values of 0.046 mg ml^-1^ and 769 Umg^-1^[33]. Looking at the kinetic properties of the other recently studied amylolytic enzymes, it is therefore suggested that both the hydrolases from *A. fumigatus* KIBGE-IB33 could be used for industrial starch saccharification purpose. Both partially purified enzymes were used for bioethanol production.

**Table 2:** **Purification profile of starch hydrolyzing enzymes produced from *Aspergillus fumigatus *** **KIBGE-IB33**

**Steps**	**Total volume (ml)**	**Total enzyme units (kU)**	**Total protein (mg)**	**Specific activity (kU mg**^**-1**^**)**	**Fold purification**
**Alpha amylase**					
Crude	500	5500	2500	2.2	1.0
(NH_4_)_2_SO_4_ Precipitation	30	1950	210	9.2	4.1
**Amyloglucosidase**					
Crude	500	229500	2500	91.8	1.0
(NH_4_)_2_SO_4_ Precipitation	30	82500	210	393	4.2

**Figure 4:**
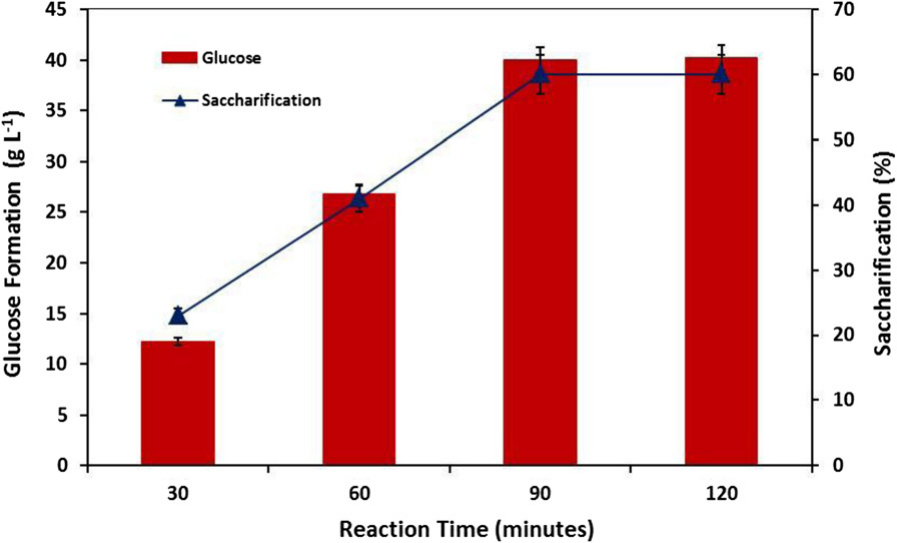
Percent saccharification of cassava starch and glucose formation at different reaction time intervals.

Conversion of starchy materials into ethanol is an intricate process and several attempts have been made to produce bioethanol in commercially feasible quantities and to easily scale-up the methodology used. Cassava starch is a complex molecule containing amylose and amylopectin and for the production of bioethanol, first the starch molecules must be hydrolyzed into more simple sugars. Some pretreatment techniques including hot water and steam explosion treatment, alkaline and solvent pretreatment, acid hydrolysis and enzymatic degradation for the breakdown of complex starch molecule into simpler sugars have also been studied [34-37]. More recently, a new pre-treatment technique known as popping pre-treatment have gained attention for the hydrolysis of starchy feedstock [38]. However, enzymatic degradation using different hydrolases is mostly preferred because during acid hydrolysis the percent conversion of starch into reducing sugars is low as compared to the enzymatic degradation [39-41]. With the progression and advancement in enzymology, amylolytic enzymes are now preferable over conventional methods because enzymatic treatments lead towards high yield of glucose with reduced energy consumption. Therefore, in the current study gelatinized cassava starch was liquefied using α-amylase and was further saccharified by means of amyloglucosidase. However, before breaking starch into simple fermentable sugars, the time required for both the processes to occur effectively was also analyzed by incubating the gelatinized starch slurry with both partially purified amylolytic enzymes for different time intervals. Glucose was the main end-product which is required for production of bioethanol, therefore the concentration of glucose formation as well as percent saccharification was monitored throughout this study. Gelatinized cassava starch was mixed with partially purified α-amylase (9.2 kUmg^-1^) and amyloglucosidase (393.0 kUmg^-1^). It was observed that as the reaction time increases, the formation of glucose (40.0 gL^-1^) as well as percent saccharification (60.0%) also increased up to 90.0 minutes and beyond that both parameters become constant (Figure 4). This glucose containing mixture was further used for the production of ethanol. Efficiency of enzymatic liquefaction and saccharification also depends upon optimum enzyme activity as well as the purity of amylolytic enzymes as crude enzyme takes longer time period to completely hydrolyze starch molecule into glucose as compared to the purified enzyme (Table 3). This percent saccharification (60.0%) could also be further augmented by either improving the purity of enzyme or by incorporating other hydrolyase (xylanases, pectinases or cellulases) along with these amylolytic enzymes [42,43]. As reported earlier further increase in percent saccharification could also be achieved if the starch slurry was autoclaved before addition of amylolytic enzyme [44]. Similarly, Aggarwal *et al.*[45] and Soni *et al.*[46] have also discussed about the role of the purity level of amylolytic enzymes during starch hydrolysis. In the same way, Shanavas *et al.*[20] have also previously analyzed the effect of reaction time on saccharification of cassava starch and have obtained maximum percent saccharification after 30.0 minutes of incubation followed by slight increase when using commercially available starch hydrolyzing enzymes. On the contrary, Aggarwal *et al.*[45] reported maximum percent saccharification using crude amylolytic enzymes after 24 hours of incubation time. Very recently, Gohel *et al*. [47] used simultaneous saccharification and solid state fermentation for the production of ethanol using Indian sorghum feedstock and also incorporated acid fungal protease instead of urea for better ethanol yield.

**Table 3:** Optimized conditions for starch hydrolysis in the presence of crude and partially purified amylolytic enzymes

Enzyme*	**Enzyme Units (kU mg**^**-1**^**)**	Starch** (gL^**-1**^**)**	**Function**	**Time (minutes)**	**Temperature (°C)**	**pH**
Alpha Amylase (Crude)	2.2	20.0	Liquefaction	120	65	5.0
Alpha Amylase (Partially Purified)	9.2	20.0	Liquefaction	15	65	6.0
Amyloglucosidase (Crude)	91.8	20.0	Saccharification	360	60	5.0
Amyloglucosidase (Partially Purified)	393.0	20.0	Saccharification	90	60	5.0

*Both enzymes were produced by *Aspergillus fumigatus* KIBGE-IB33.

**Cassava starch.

A large number of microbes including bacteria, yeast and fungi are capable of producing ethanol from fermented sugars [48]. Among them *S. cerevisiae* is widely used for production of bioethanol because it is not only able to produce high amount of ethanol but it can also tolerate and survive higher concentrations of ethanol formed in the medium [49,50]. However, most of the yeast strains are not capable to directly ferment complex starchy materials [48]. Therefore, in the current study *S. cerevisiae* was used for the production of ethanol from glucose which was earlier formed by the action of fungal amylolytic enzymes. Table 4 summarizes the results of bioethanol production after 24 and 48 hours of yeast fermentation and it was noticed that the maximum percent yield of bioethanol (84.0%) was obtained after 48 hours. After 48 hours, the fermented medium was distilled and the percent yield became 53.0%. The concentration and purity of the distilled ethanol was also analyzed using gas chromatography (GC).

**Table 4:** **Production of bioethanol using *Saccharomyces cerevisiae***

**Incubation time (Hour)**	**Glucose concentration (gL**^**-1**^**)**	**Ethanol (%)**	**Theoretical yield (g)**	**Actual yield (g)**	Yield*^* ^**(%)**
		**Before distillation**			
24	40.0	36.0	2.04	1.44	70
48	40.0	43.0	2.04	1.72	84
		**After distillation**			
48	40.0	27.0	2.04	1.08	53

*Mass of ethanol formed per mass of glucose consumed.

Several techniques including direct fermentation, simultaneous saccharification, simultaneous non-thermal saccharification, ultrasound assisted treatment and solid-state fermentation have been studied previously using different starchy materials and microbial sources for the production of bioethanol [20,21,51-56]. Along with this ethanol has also been produced by repeated batch culture through immobilization of *S. cerevisiae* and *S. pastorianus* IFO0751 on calcium alginate and porous cellulose carriers, respectively [57,58]. Nikolic *et al*. [54] used ultrasound-assisted treatment for direct conversion of corn meal into bioethanol but the cost related to this method in amount of energy consumption is very high. Beside this, the pretreatment of multiple biomass or starch flour will also add extra budget that will eventually affect the feasibility of the bioethanol. The attempt made in the current study by consuming commercially available cheap cassava starch along with saccharification by synergistic effect of fungal amylolytic enzymes had revealed that this two-step based method can be used to achieve higher yields of bioethanol. Further, the process cost can also be reduced by using other inexpensive starchy materials or by establishing pilot programs that will scrutinize the actual feasibility and sustainability of the overall process developed.

## Conclusions

In conclusion, an improved and effective enzymatic saccharification of inexpensive cassava starch using amylolytic enzymes from *A. fumigatus* KIBGE-IB33 was developed. The glucose obtained after enzymatic degradation was utilized for the bioethanol production using *S. cerevisiae*. Dual systematic enzyme conversion has advantages in terms of reduced energy consumption as well as increased production of fermentable sugar to achieve maximum bioethanol yield as compared to other processes. In addition, the process developed is more rapid as compared to the previously conducted studies using liquefaction and saccharification of cassava starch.

## Methods

### Reagents

All reagents were of analytical grade and were obtained from commercial sources. Sago and cassava starch were purchased from local market, Karachi, Pakistan. Peptone (Oxoid, England), yeast extract (Oxoid, England), ammonium sulfate (Serva, Germany) and dipotassium hydrogen phosphate (Serva, Germany) were purchased from a local vendor. Whereas, magnesium sulfate, sodium hydroxide, sodium carbonate, copper sulfate, sodium potassium tartarate, sulfuric acid and potassium dichromate were acquired from Scharlau (Spain). Other chemical used including 3′, 5′- dinitrosalicylic acid (DNS) was purchased from BDH Chemicals (USA) and anthrone from MP Biomedicals (France) while, soluble starch and folin ciocalteu reagent were purchased from Merck (Germany).

### Isolation and identification of filamentous fungi

The natural fungal isolates used in the current study were isolated from different soil samples that were collected aseptically from diverse vegetative fields located in Karachi, Pakistan. All the isolates were obtained after serial platting on potato dextrose agar (PDA) at 30°C for 05 days according to the standard protocols. PDA medium consist of (gL^-1^): Boiled potato extract, 300.0 ml; dextrose, 20.0 g and agar, 16.0 g. Initially, 07 different fungal species were isolated from different samples. Among them 05 filamentous fungi were selected and characterized based on colonial morphology, 18S rDNA sequence cataloging and microscopic analysis using lactophenol blue staining method [59,60]. After 18S rDNA gene analysis and sequencing, the sequences were analyzed by similarity search using BLAST (http://www.ncbi.nlm.nih.gov/BLAST/) and were submitted to NCBI GenBank database. The confirmed sequences received the following GenBank accession numbers: KF905648*,* KF905649*,* KF905650*,* KF905651*,* and KF905652 for *A. fumigatus* KIBGE-IB33, *A. flavus* KIBGE-IB34, *A. terreus* KIBGE-IB35, *A. niger* KIBGE-IB36 and *A. versicolor* KIBGE-IB37, respectively. All of these isolates were tested for the amylolytic enzyme production based on starch-iodine plate method. All fungal isolates were plated on starch medium plates containing (gL^-1^): cassava starch, 10.0; yeast extract, 10.0; peptone, 10.0; K₂HPO₄, 1.0; and MgSO_4_.7H_2_O, 1.0. The cultures were incubated at 30°C for 05 days. After incubation the plates were flooded with potassium-iodide solution for the detection of amylolytic activity. Isolates were selected based on clear halo-zone around the fungal growth. All isolates were preserved on PDA slants at 4°C for further analysis and were sub-cultured routinely. Purified *Sacchromyces cerevisiae* (baker’s yeast) was purchased from the local market, Karachi, Pakistan and was grown and maintained in YPD medium (gL^-1^:yeast extract,10.0; Bacto-peptone, 20.0 and glucose, 20.0).

### Inoculum preparation for seed culture

Total viable spores were calculated in order to prepare fungal inoculum. For this purpose spores were transferred using sterile needle from a 05 day old fungal culture grown on PDA slant and re-suspended in 10.0 ml sterile distilled water containing 0.1% Tween-20. Each suspension was serially diluted up to 10^-5^ in order to make homogenous spore suspension of 10^6^ to 10^8^spores ml^-1^).

### Medium used for the production of amylolytic enzymes

All the selected filamentous fungi were tested for the production of α-amylase and amyloglucosidase in the presence of starch (cassava) under batch conditions using submerged fermentation technique. Production of α-amylase, amyloglucosidase and glucose was monitored at different time intervals (02 to 07 days). Basal medium used for the production of α-amylase and amyloglucosidase consists of (gL^-1^): Cassava starch, 20.0; yeast extract, 10.0; peptone, 10.0; K_2_HPO_4_, 1.0; and MgSO_4_.7H_2_O, 1.0. Initial pH of the medium was adjusted at 7.0 before sterilization at 121°C for 15 minutes. Fresh seed culture (10.0 ml) was inoculated in 90.0 ml production medium and incubated at 30°C for 03 days under static and anaerobic conditions. It was then further transferred into 900.0 ml medium and incubated at 30°C up to 07 days. The fungal spores were harvested by centrifuging the fermented broth at 40248 × *g* for 15 minutes at 4°C. The supernatant was filtered using 0.45 μ filter under vacuum. The cell free supernatant containing the amylolytic enzymes was stored at -20°C for further analysis. All the experiments were conducted in triplicates.

### Optimization of physicochemical parameters for maximum enzyme yield

For the enhanced production of amylolytic enzymes, different inducing substrates (carbon sources) and fermentation time were optimized. For this purpose seven different carbon sources including sago starch, soluble starch (potato), cassava starch, wheat starch, wheat bran, rice bran and sugarcane bagasse were used in the concentration of 20.0 gL^-1^. *A. fumigatus* KIBGE-IB33 was incubated for different time intervals ranging from 02 to 07 days at 30°C under static and anaerobic condition for the selection of optimum fermentation time. Enzyme titer in terms of specific activity and glucose formation were monitored in triplicate.

### Partial purification of amylolytic enzymes

The cell free supernatant containing α-amylase and amyloglucosidase was precipitated using salt precipitation method. For this purpose, salt gradient precipitation technique was employed ranging from 20.0% to 80.0% saturation using ammonium sulfate. 20% salt was incorporated gradually in CFF with continuous stirring at 4°C and was equilibrated for 18 hours. The precipitates formed were centrifuged at 40248 × *g* for 10 minutes at 4°C and were dissolved in citrate buffer (50.0 mM, pH-5.0). In the next run, again the 20% salt saturation was performed using the same supernatant up to 80% and every time the precipitates were equilibrated for 18 hours at 4°C. During each saturation range, the precipitates were monitored and calculated for both enzymes unit in terms of kU mg^-1^ of protein. The saturation level at which both hydrolyase were precipitated out with maximum unit was selected (40%).

### Enzyme assay and total protein estimation

Enzyme activity of α-amylase and amyloglucosidase was estimated using DNS [61] and GOD-PAP method [62,63], respectively. One unit of α-amylase is defined as the “amount of enzyme that liberates 1.0 mM of maltose per minute under standard assay condition”. Whereas, one unit of amyloglucosidase is defined as the “amount of enzyme that liberates 1.0 mM of glucose per minute under standard assay condition”. The specific units of both amylolytic enzymes are expressed in terms of kilo units per mg of protein (kU mg^-1^). Total protein was calculated using Lowry’s *et al*. [64] method with bovine serum albumin as standard.

### Production of bioethanol

Bioethanol was produced using glucose, which was obtained after hydrolysis of starch (cassava) using partially purified amylolytic enzymes. Partially purified α-amylase and amyloglucosidase (30.0 ml) was amalgamated in 2.5 liters of pre-gelatinized cassava starch slurry (20.0 gL^-1^) which was prepared in citrate buffer (pH-5.0, 50.0 mM). The reaction mixture was kept at 65°C for liquefaction (15.0 minutes) and at 60°C for saccharification (90.0 minutes). The reaction was terminated by boiling the reaction mixture for 10 minutes. The volume was reduced up to 2.5 folds and the liberated glucose was detected before and after concentrating the reaction mixture. However, for optimized liquefaction and saccharification condition, the reactions were monitored from 15 minutes up to 08 hours with an interval of 15 minutes. Percent saccharification of cassava starch was calculated as followed:

$$\mathrm{Saccharification}\;\left(\%\right)=\frac{\mathrm{Glucose}}{\mathrm{Substrate}}\times 100$$

In this concentrated hydrolyzate, yeast extract and peptone were incorporated in the concentration of 3.0 gL^-1^ and 10.0 gL^-1^, respectively and the pH was adjusted up to 7.0. *S. cerevisiae* (2.0%) was cultured in this medium anaerobically at 30°C up to 48 hours. After 48 hours, ethanol was collected through distillation and the distillate was analyzed for the detection of ethanol. Percent yield of bioethanol was calculated as followed:

$$\begin{array}{ll} \mathrm{Ethanol}\;\mathrm{Yield}\;\left(\%\right)= & \frac{\mathrm{Actual}\;\mathrm{Ethanol}\;\mathrm{Produced}\;\left(g\right)}{\mathrm{Theoritcal}\;\mathrm{Ethanol}\;\mathrm{from}\;\mathrm{Sugar}\;\mathrm{Consumed}\;\left(g\right)}\\ & \times 100 \end{array}$$

### Analytical method for bioethanol analysis

For the determination of bioethanol concentration, the distillate was analyzed using Caputi *et al*. [65] method. The fermented broth was distilled using a Heating Mantle (Barnstead-Electrothermal, Thermo Scientific) at 78°C along with a quick fit distillation apparatus equipped with a Lie-big condenser and the in-let cold water attached to a chiller. All the experiments were run independently in triplicate and the results presented are the mean of three values.

### Determination of bioethanol concentration by gas chromatography (GC)

Bioethanol concentration was also verified using gas chromatography system (GC17A, Shimadzu, Japan) equipped with flame ionization detector (FID). Column used was TRB-5 (30 × 0.25 mm × 0.25 μm) with nitrogen as a carrier gas (20 cmsec^-1^). The temperature of the detector and the injector were kept at 200°C and 130°C, respectively. The split ratio was 100:1 and the peak area of the compound was integrated against an external standard of absolute ethanol.

### Physico-chemical characteristics of cassava starch

The cassava starch used in this study was purchased from the local market in Karachi, Pakistan. For the determination of total sugar anthrone method was used [66] whereas, reducing sugar was detected using DNS method [61]. Total protein was performed using Lowry’s *et al*. [64] method. Glucose content was estimated using GOD-PAP method [62,63]. Moisture content was calculated using standard drying method at 105°C until the weight become constant. Amylose and amylopectin fractions were calculated by iodometric method as suggested previously [67].

## Abbreviations

CFF: Cell free filtrate; DNS: 3′, 5′-dinitrosalicyclic acid; FID: Flame ionization detector; GC: Gas chromatography; gL^-1^: Grams per liter; GOD-PAP: Glucose oxidase per oxidase method; kU mg^-1^: Kilo units per milligrams; mM: Milli moles; PDA: Potato dextrose agar.

## Competing interests

Its publication is approved by all authors and they do not have any conflict of interest regarding any financial, personal or other relationships with any other people or organizations.

## Authors’ contributions

SP carried out the major experimental work. AA supervised in acquisition of laboratory data and interpretation of data along with finalizing the manuscript. SI participated in the analytical analysis of bioethanol. NNS performed enzyme purification. SAQ designed the project and gave the final approval of the manuscript. All authors have read and approved the final manuscript.
